# A Model of Loggerhead Sea Turtle (*Caretta caretta*) Habitat and Movement in the Oceanic North Pacific

**DOI:** 10.1371/journal.pone.0073274

**Published:** 2013-09-05

**Authors:** Melanie Abecassis, Inna Senina, Patrick Lehodey, Philippe Gaspar, Denise Parker, George Balazs, Jeffrey Polovina

**Affiliations:** 1 Joint Institute for Marine and Atmospheric Research, University of Hawaii, Honolulu, Hawaii, United States of America; 2 Collecte Localisation Satellite, Ramonville-Saint-Agne, France; 3 Pacific Islands Fisheries Science Center, National Ocean and Atmosphere Administration, Honolulu, Hawaii, United States of America; University of Wales Swansea, United Kingdom

## Abstract

Habitat preferences for juvenile loggerhead turtles in the North Pacific were investigated with data from two several-year long tagging programs, using 224 satellite transmitters deployed on wild and captive-reared turtles. Animals ranged between 23 and 81 cm in straight carapace length. Tracks were used to investigate changes in temperature preferences and speed of the animals with size. Average sea surface temperatures along the tracks ranged from 18 to 23 °C. Bigger turtles generally experienced larger temperature ranges and were encountered in warmer surface waters. Seasonal differences between small and big turtles suggest that the larger ones dive deeper than the mixed layer and subsequently target warmer surface waters to rewarm. Average swimming speeds were under 1 km/h and increased with size for turtles bigger than 30 cm. However, when expressed in body lengths per second (bl s^−1^), smaller turtles showed much higher swimming speeds (>1 bl **s** ^−**1**^) than bigger ones (0.5 bl s^−1^). Temperature and speed values at size estimated from the tracks were used to parameterize a habitat-based Eulerian model to predict areas of highest probability of presence in the North Pacific. The model-generated habitat index generally matched the tracks closely, capturing the north-south movements of tracked animals, but the model failed to replicate observed east-west movements, suggesting temperature and foraging preferences are not the only factors driving large-scale loggerhead movements. Model outputs could inform potential bycatch reduction strategies.

## Introduction

Bycatch of non-target species, and especially of endangered species, has long been recognized as an issue and there is a broad consensus, and even mandates in some countries, to minimize bycatch levels [1]. Identifying critical habitat and quantifying relationships between movements and environmental variables has become a common approach for spatial management of commercial or endangered species [2]–[4].

Loggerhead sea turtles (*Caretta caretta*) are distributed in tropical and temperate areas of each ocean basin and occupy pelagic, coastal and terrestrial habitats during their life cycle. Their only known nesting areas in the Pacific are located in Japan, Australia, and New Caledonia [5], [6]. During their juvenile phase, loggerheads spend years, and probably decades in the open ocean. Some juvenile populations are also found in coastal regions. One such area is located in Baja California, Mexico, where turtles from the Japanese nesting regions occur [7]–[9]. Most likely, loggerheads possess two different but potentially overlapping foraging strategies, yielding some to spend their entire juvenile phase in the open ocean, and some others in coastal areas [10], [11]. When they reach sexual maturity, the adults then undertake long migrations towards the nesting beaches, averaging about 2 to 3 years, but may return more or less frequently depending on foraging success. There is uncertainty on the age at maturity of loggerheads, but estimates range between at least 10 and 45 years – this late maturity heightens concerns for their conservation [12]–[14].

Loggerheads were relisted as endangered in the North and South Pacific by the U.S. National Marine Fisheries Service and the U.S. Fish and Wildlife Service in September 2011 under the U.S. Endangered Species Act and have been listed as endangered by the IUCN (International Union for Conservation of Nature) since 1996. Loggerheads face various anthropogenic threats at every stage of their life cycle, such as loss or alterations of nesting beaches, directed takes, ingestion of marine debris, environmental contamination, diseases, and interactions with various fisheries (pound net, gillnet, trawl and longline fisheries) [15]. One particular threat juvenile loggerheads face during their oceanic phase is incidental takes in pelagic longlines, especially shallow longlines targeting swordfish. Bycatch in the gillnet and trawl fisheries in coastal areas has been estimated to be equally high or higher than longline bycatch [16] and the Japanese pound net fishery may kill over 1000 loggerheads every year [17]. These fisheries affect mostly adult and sub-adults [16], [18]. This paper focuses on bycatch of juveniles in the North Pacific longline fisheries.

Considerable efforts to reduce turtle bycatch in these fisheries have been expended, in particular in the United States, following temporary closures of the Hawaii-based fishery from 2000–2004 and the enactment of new regulations. Gear and fishing method changes brought about by the new regulations have reduced turtle and seabird bycatch rates [19]. Substantial reductions in interactions with protected species have been observed [20]–[23]. In the Hawaii-based longline fishery, there has been 100% observer coverage since 2004, and the shallow-set fishery closes if more than 34 loggerhead sea turtles are caught in any calendar year (the annual catch limit was raised by the National Marine Fisheries Service – NMFS, from 17 to 34 on Nov. 5, 2012). However, bycatch mortality continues, and could possibly increase with the higher catch limit. And some studies suggest that there may have been a transfer of the bycatch mortality to non-US fleets as a result of strict regulations in the US fisheries [24].

Further reductions of loggerhead takes in the longline fishery may be achieved if longline fisheries target swordfish in places and during times when loggerhead turtles are not occupying the same habitat as swordfish [23]–[28]. To achieve this will require a better understanding of the pelagic habitats of swordfish and loggerhead turtles. The pelagic habitat of loggerhead turtles in the North Pacific has been fairly well described as a result of considerable electronic tracking [10], [29]–[32]. However, all these studies rely on the same extensive dataset. Few studies of oceanic juvenile loggerheads exist in the North Pacific.

A statistical analysis between sea surface temperature (SST), loggerhead turtles tracks in the fishing grounds of the shallow-set Hawaii longline fishery and the locations of high occurrence of bycatch within that fishery, was used to design a first index of high probability of presence of turtles which was developed by the NOAA Pacific Islands Fisheries Science Center (PIFSC). The index, called TurtleWatch, is displayed on maps and distributed weekly to fishermen [23], advising them to avoid areas characterized by SST between 17.5 and 18.5°C (www.pifsc.noaa.gov/eod/turtlewatch.php), which corresponds to the temperature range in which 50% of the fisheries interactions with loggerheads occurred between 1994 and 2006. While gear and fishing regulations have greatly reduced the number of turtle bycatch by the fishery, this approach can provide additional information to attempt to minimize turtle bycatch.

The main focus of this paper is to use satellite tracking data to adapt and evaluate the feeding habitat definition, initially developed within a spatial population dynamics model for tropical tunas, to predict more precisely the feeding habitat and movements of juvenile loggerhead turtles in the North Pacific. This approach could also be applied to other species of marine turtles or to other marine organisms whose temperature preference or foraging strategies are well defined.

## Materials and Methods

### Tagging and Tracks Processing

Forty electronic tags were placed by scientific observers on loggerhead turtles caught as bycatch in the Hawaii-based longline fishery from 1997 to 2000 (hereafter referred to as longline bycatch releases or LL bycatch releases); 184 loggerhead turtles were reared in captivity for research purposes of pelagic tracking at the Port of Nagoya Public Aquarium, in Minoto-ku, Japan, and released in the ocean in several different locations during 8 deployments between 2003 and 2007 (hereafter referred to as Japanese releases. See Tables 1 and 2).

**Table 1:** Releases from Hawaii-based longline vessels.

id	datedeployed	longitude	latitude	SCL^1^	# days out
24181	01/23/97	190.21 E	28.708N	44.5	55
19580	02/02/97	196.7 E	29.480N	52	115
19585	02/15/97	199.01 E	29.782N	41	90
19582	03/17/97	205.57 E	30.863N	62	136
24184	03/30/97	199.42 E	26.160N	73	42
19587	04/10/97	191.01 E	26.743N	73.6	13
19581	04/20/97	205.32 E	29.232N	53.7	12
19586	04/22/97	203.42 E	28.758N	81	178
24182	09/11/97	228.93 E	37.722N	45	67
19599	01/06/98	216.97 E	33.567N	45.5	206
19598	01/07/98	217.46 E	34.383N	48	191
19594	02/07/98	205.35 E	30.567N	58	103
24185	02/07/98	204.95 E	30.533N	61	71
7298	03/10/98	190.35 E	28.967N	74	0
7299	03/15/98	190.63 E	28.533N	73.5	0
19591	04/06/98	201.76 E	27.911N	76	1
19590	08/26/98	196.7 E	36.438N	57.7	106
19608	08/26/98	197.36 E	36.288N	58	167
19601	10/18/98	195.22 E	37.717N	52.5	41
19606	10/20/98	220.4 E	38.477N	59.1	161
19604	11/02/98	198.1 E	36.667N	62.5	51
25360	12/05/98	197.53 E	34.250N	67	1
24189	12/05/98	197.53 E	34.250N	59	1
24190	12/10/98	223.9 E	34.230N	56.5	6
25359	12/23/98	210.02 E	33.642N	57.5	211
19605	01/04/99	207.72 E	32.183N	46	0
25358	01/04/99	207.72 E	32.183N	54	1
25361	01/30/99	206.35 E	32.038N	53.5	0
19602	01/31/99	203.68 E	24.767N	83	51
19597	02/03/99	215.32 E	31.917N	60	1
24179	02/03/99	206.18 E	32.028N	52.5	131
22174	12/14/99	209.08 E	32.921N	51.5	271
22173	01/17/00	216.71 E	32.772N	62	72
24188	01/31/00	215.52 E	31.833N	54	0
22152	02/03/00	190.72 E	32.686N	67	157
22172	02/12/00	221.45 E	32.256N	55	49
22150	03/05/00	213.45 E	31.133N	60	597
22153	03/07/00	213.29 E	31.065N	56	246
24747	05/30/00	205.23 E	24.955N	83	138
22534	08/19/00	226.4 E	35.794N	61	177

1 Straight Carapace Length, in cm.

**Table 2:** Releases of turtles reared at the Port of Nagoya Public Aquarium.

date deployed	longitude	latitude	# deployed^1^	SCL^2^	# days out
04/24/03	140.166E	34.643N	7	38.9–59.4	67–565
11/28/03	140.233E	34.867N	18	26.2–56.0	48–1270
04/23/04	141.122E	35.431N	13	25.6–64.8	27–626
11/19/04	140.590E	34.867N	26	28.4–35.3	85–462
05/04/05	176.617E	32.667N	44	29.6–38.4	229–1368
07/30/05	136.900E	33.950N	16	39.2–47.0	0–438
10/27/06	176.832E	32.852N	35	23.3–30.2	47–493
09/24/07	140.590E	34.867N	25	23.6–28.2	62–465

1 # of tags deployed.

2 Straight Carapace Length, in cm.

Turtles were outfitted with satellite transmitters attached to the carapace using the procedures described in [33]. They were equipped with Telonics (Mesa, AZ, USA) model ST-18, ST-19, ST-24, and Wildlife Computers (Redmond, WA, USA) model SDR-T10, SDR-T16, or SPOT 3/4/5 Argos-linked satellite transmitters. Tracking data were collected through the ARGOS system by the NOAA PIFSC, Marine Turtle Research Program, Honolulu, Hawaii.

To filter out Argos location errors, all tracks were processed the same way as in [34]. First, all locations resulting from velocities greater than 10 km/h were removed. Then, to remove additional artificial “spikes” from the data, an Epanechnikov filter [35] was applied with a 2-day window, centered on each individual location, using the *lpepa* function of the *lpridge* package in the R environment [36]. The points providing the 5% largest differences between filtered and observed locations were removed. The original track without these extreme locations was then resampled from a few locations a day (average number of transmissions = 2.6/day) to a location every 3 hours. Finally, a second Epanechnikov filter was applied every 3 hours with a 2-day window to smooth the track.

### Ethics Statement

Tagging and release of turtles from the Hawaii-based longline vessels were done by shipboard observers working with the NOAA/NMFS Observer Program under the U.S. ESA permit #1190. All other animal care and permitting needs for this study were accomplished by the Port of Nagoya Public Aquarium, in full compliance with the requirements and approval of the government of Japan, of which the Aquarium is an entity. Permission to use this data was granted by Dr. Makoto Soichi under a partnership between the Aquarium and the Pacific Islands Fisheries Science Center. All permissions to release the turtles in international waters and Japanese waters were obtained.

**Size, temperature preference, and swimming speed.**

The temperature preference and swimming speed of animals were estimated from the tracks using 0.1°- weekly Pathfinder-GAC satellite SST data (http://www.nodc.noaa.gov/SatelliteData/pathfinder4km/), provided by the NOAA-Coastwatch program, and filtered 1°×1°×5d ocean surface currents data, downloaded from the NOAA-OSCAR website (http://www.oscar.noaa.gov). Both data sets were chosen for their coverage of the entire tracking period (1997 to 2008). Values were extracted along each processed track using the Generic Mapping Tools (http://gmt.soest.hawaii.edu/).

The change in curved carapace length (CCL) along the tracks was estimated using recently published results on loggerhead turtles growth rates [14] to account for the change in size, especially during protracted tracking periods: growth rate (cm/yr CCL) = −10.6 * log10(CCL) +21.5. Since turtle sizes were measured in straight carapace length (SCL) on release, CCL was converted to SCL using the relationship in [11]:

SCL = 0.369+0.932*CCL.

To evaluate the change in temperature habitat with size, a generalized additive model (GAM, [37]) was built using the *mgcv* (version 1.7–13) library in R [38] to study the relationship between SST along the tracks and size, while accounting for other potential sources of variability in SST, such as the interannual (year) and seasonal (month) variations, as well as latitude and longitude.

The observed velocity of a swimming animal (**V**g) is the sum of the animal’s own velocity (**V**) and the velocity of the current (**V**c). To study the turtles active movements, and separate swimming from drifting, ocean currents were removed from the tracks (**V** = **V**g–**V**c, [34]). Loggerhead turtles spend 90% of their time within the first 5 m of the water column [32]; therefore, the use of OSCAR surface currents should be adequate for this analysis.

An estimate of maximum sustainable speed (MSS) was then computed as the monthly 90th percentile of the animals’ active movement speed and was converted in body lengths per second (bl s-1) by dividing it by the animal’s monthly estimated SCL. The 90th percentile was chosen arbitrarily as a proxy for MSS.

### Habitat and Movement Modeling

To model loggerhead turtle habitat and movements, we used MOVEMOD, a simplified non age-structured version of the SEAPODYM (Spatial Ecosystem And Populations Dynamics Model) model, forced by a realistic 3D physical ocean and by primary production estimated by satellite.

The Mercator-Ocean GLORYS-1 (GLobal Ocean ReanalYsis and Simulations) reanalysis was used to provide temperature and ocean currents forcing fields, in conjunction with net primary production data derived from ocean color satellite data (http://www.science.oregonstate.edu/ocean.productivity/). GLORYS-1 is an eddy-permitting global ocean reanalysis produced for the 2002–2007 period with the ocean general circulation model configuration ORCA025 NEMO [39] at a spatial resolution of ¼°. Its results are available at a daily time step. The assimilation method is based on a reduced order Kalman filter (SEEK formulation, [40]) adapted to this configuration [41]. Because satellite (SST and altimetry) and in situ data are assimilated in this ocean reanalysis; predicted fields of temperature and currents are coherent with those of primary production derived from ocean color data, using the VGPM model [42]. To be used as forcings for the SEAPODYM model, both GLORYS-1 outputs and primary production data were interpolated on a regular 0.25°×0.25° grid with a 6-day time step.

### SEAPODYM

SEAPODYM was originally developed to model the age-structured spatial dynamics of tropical tuna populations in pelagic ecosystems in interaction with their environment [43]–[47]. It uses physical-biogeochemical environmental fields to simulate the upper trophic levels of marine ecosystems organized in two groups: the predator species (e.g. tuna, billfish, or turtles) and their prey species of the mid-trophic levels (i.e., micronekton). SEAPODYM is built with a three-layer structure: epipelagic between the surface and one euphotic depth (*Z_eu_*), mesopelagic between 1 *Z_eu_* and 3 *Z_eu_*, and bathypelagic between 3 *Z_eu_* and 7 *Z_eu_*. The micronekton functional groups are described with several components characterized by their habitat and vertical behaviour [47]. Predator movements are described by advection-diffusion equations. Diffusion is used to represent random movements (kinesis), and advection to reproduce both the transport due to currents and directed movements in response to external stimuli (taxis). Directed movements follow the gradient of a habitat index depending on temperature preference, oxygen constraints (for fish) and prey availability.

### MOVEMOD

As information is scarce on loggerhead natural mortality and reproductive dynamics, a simplified single age/size class version of the SEAPODYM model, called MOVEMOD, was used, where temperature and feeding habitats only are modeled for a given cohort. This model was adapted to air-breathing turtles by removing any oxygen constraint from the habitat definitions. As loggerhead turtles usually stay in proximity to the surface and spend most of their time in the first 30 m [32], we used GLORYS-1 temperature and horizontal currents averaged over the first 30 m of the water column instead of the euphotic depth to prevent the ocean currents and temperature signals from being diluted when averaged across the whole epipelagic layer, as defined in SEAPODYM. The only prey field available however, was the whole epipelagic component (comprising prey items that reside in the epipelagic layer during both day and night, as well as migrants from deeper layers during the night [47]. The feeding habitat index, defined as the accessibility to the forage species, is simply controlled, in the case of turtles, by a temperature preference that needs to be parameterized and by the concentration of prey. Temperature preference is modeled by a Gaussian-shape index (between 0 and 1), the mean and standard deviation of which represent the animal’s optimal temperature and temperature tolerance, respectively [45].

Then the gradient of this habitat index is used to drive the directed (advection) and random (diffusion) movements of the animals. For both types of movements, the displacement per time unit is directly dependent on the size of the individuals. Therefore, for a given size, the movement is linked to a maximum sustainable speed (MSS) expressed in body lengths per second [45]. Finally, advection and diffusion rates both also depend on the feeding habitat, so that individuals will tend to stay longer in the presence of favorable conditions (low diffusion) but will want to escape from unfavorable habitats (high diffusion) and advection will be directed towards areas of highest gradient of habitat.

### Simulations and Evaluation of the Model

Using values of temperature preference and speed assessed from the tag data analysis, the predicted habitat index was parameterized and then simulated for two different batches of releases (November 2004 and May 2005, Table 2), using the average of the turtle sizes for each batch. Those two releases were chosen because their ranges of sizes were small enough to reasonably treat all the turtles released on each day as one single age class in MOVEMOD. The habitat index predicted by the model was compared to the corresponding tracks and to the TurtleWatch index.

To study the spatial dynamics of the animals once the habitat preferences were determined, we simulated the release of a batch of *n* = 2000 “virtual” turtles in MOVEMOD, at the same locations and dates the tagging deployments occurred, and ran the model to estimate the distribution of population density over a 1-year period. We compared the simulated density distributions with the locations of the tagged turtles in every quarter to assess the validity of the modeled movements. The simulation for the May 2005 release was then repeated after switching the mechanisms of active swimming off, to compare the agreement between the modeled turtle density distribution and the observed locations at year-end in each case (passive drifting in ocean currents vs movement including advection by currents and active swimming).

## Results

**Figure 1:**
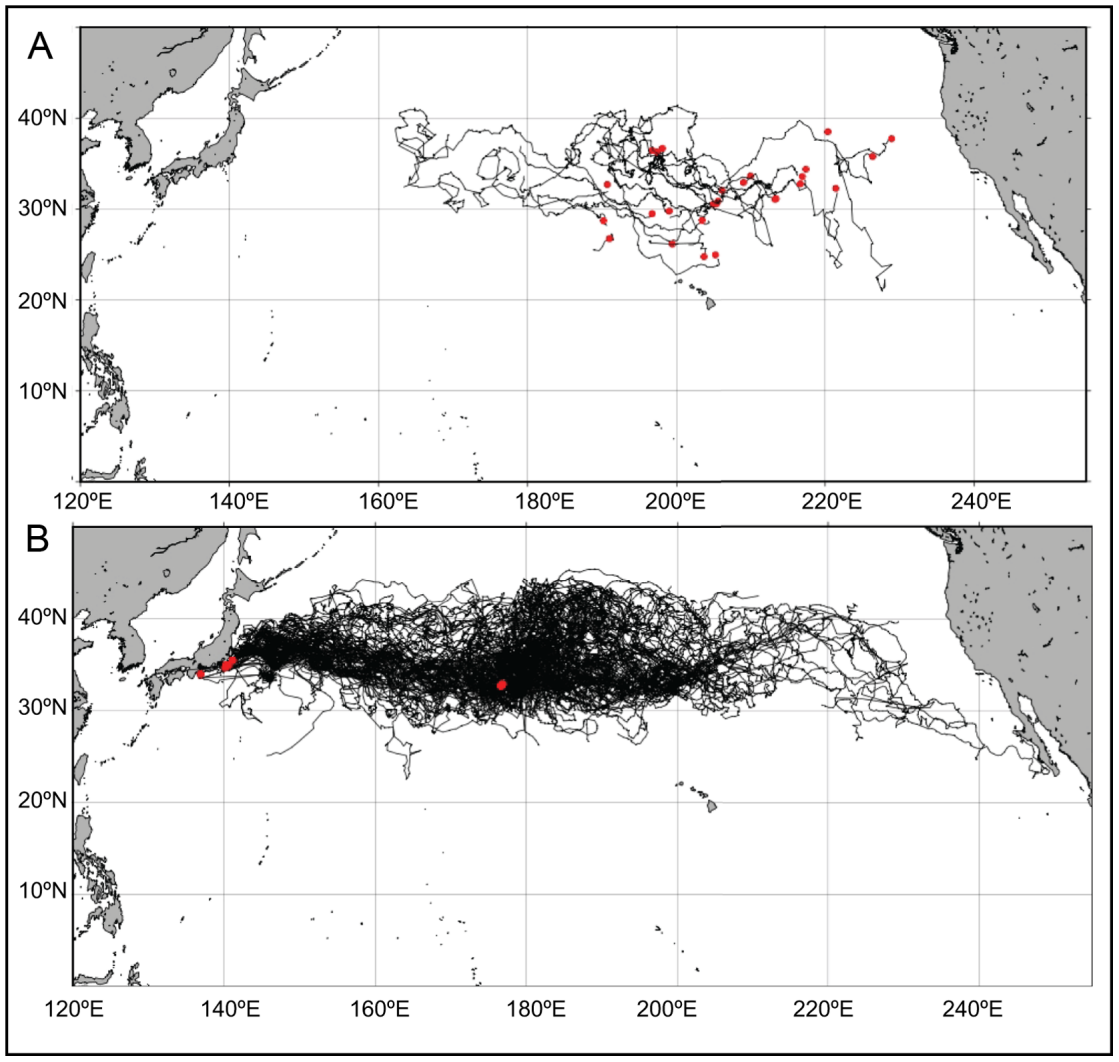
Maps of the tracks from the longline (LL) bycatch (A, n = 28) and Japanese (B, n = 160) releases. Red dots indicate release locations.

Track durations varied between 0 and 1270 days. Twelve and eight of the longline bycatch and Japanese releases, respectively, transmitted data for less than 10 days and were thus discarded from the study. Another set of 16 Japanese tracks had to be excluded because they exhibited data gaps too large (typically over 3° in longitude or latitude) for the interpolation to behave properly when resampling the tracks. Remaining processed tracks (resampled at a 3-hour interval) are shown in Fig. 1.

**Figure 2:**
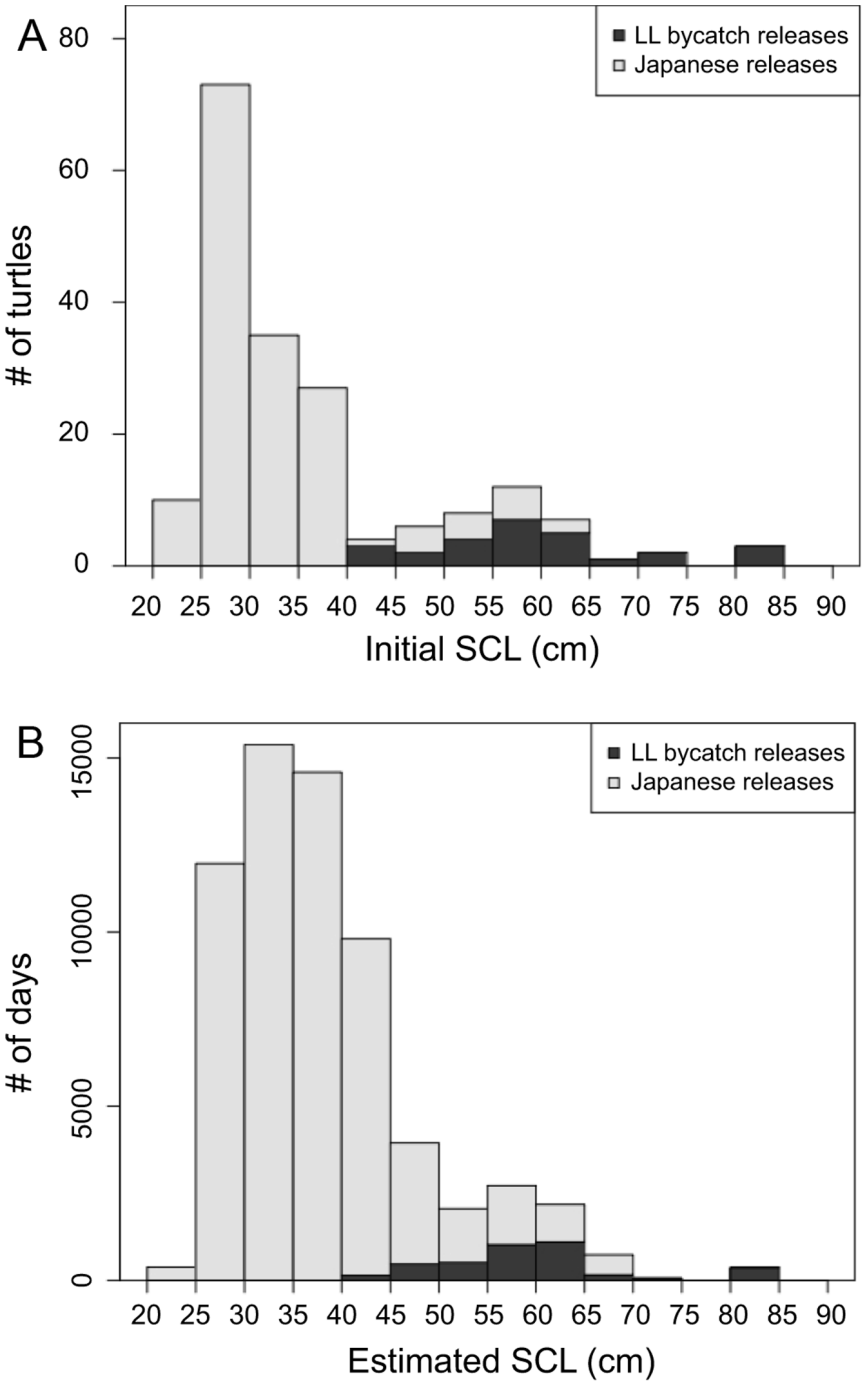
Size frequency of released turtles. At time of release (A) and estimated, accounting for growth, along the tracks (in # of days of data in each size bin, B).

The turtles tagged in the various deployments ranged between 23 and 81 cm in SCL at the time of release (Fig. 2A). The turtle sizes estimated by taking into account turtle growth along the tracks are shown in Fig. 2B. Most of the Japanese releases were smaller than 40 cm upon release, whereas all LL bycatch releases were bigger than 40 cm.

### Size Vs Surface Temperature “Preference”

**Figure 3:**
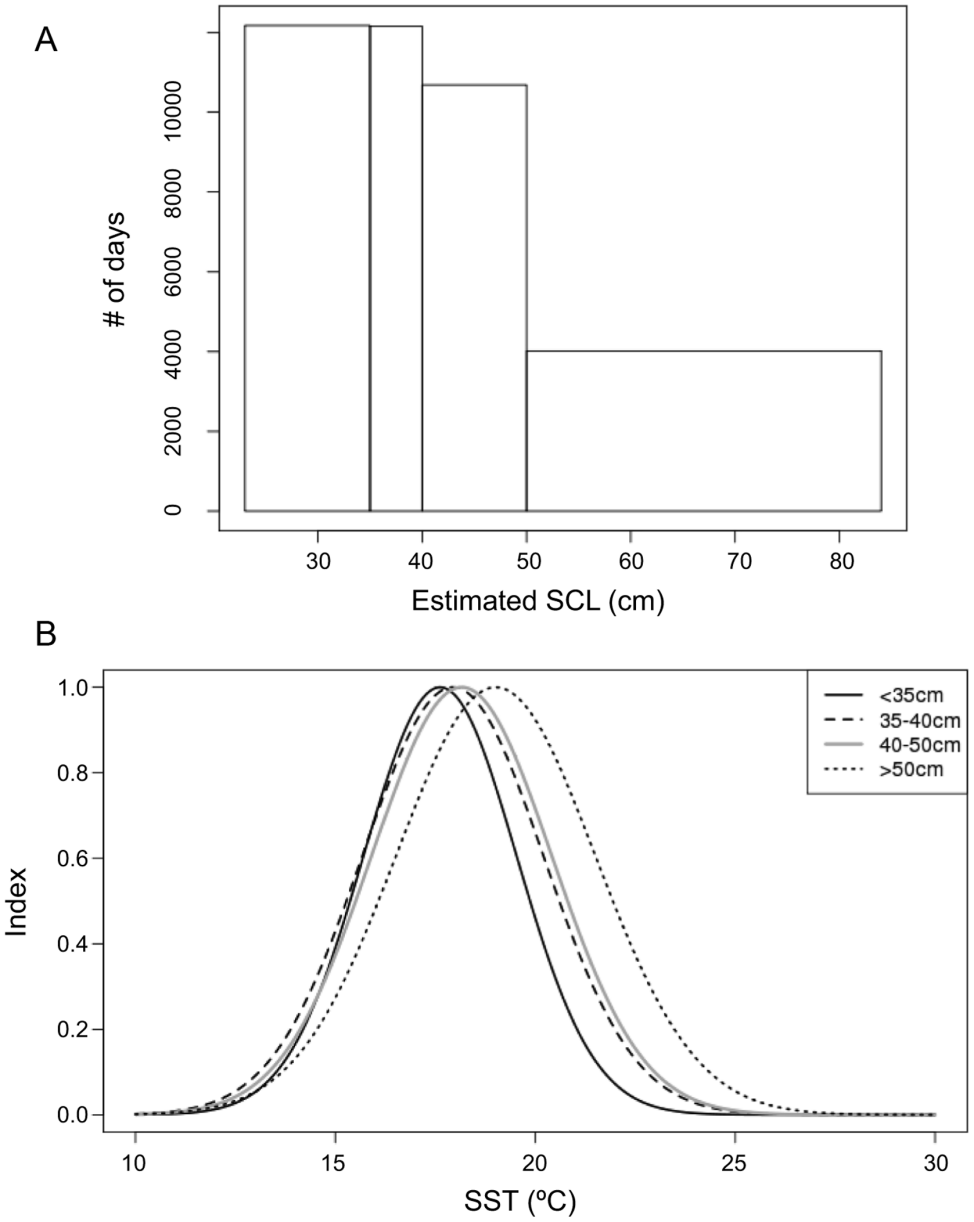
Size histogram for 4 size classes (A) and respective temperature “preference” index (B). Temperature preference index is represented as Gaussian curves.

To investigate the relationship between SST experienced along the track and the size of individuals, we grouped the data in 4 groups depending on the estimated size along the tracks (Figs. 2 & 3) and compared the mean and standard deviation of the corresponding SST values in each group. Those were plotted as Gaussian curves (Fig. 3) describing a “preference” index, between 0 and 1. The standard deviations of SST increased with size between the 4 groups, as well as the means. To avoid areas in the central Pacific Gyre from being compared with locations in the Kuroshio Current (where water temperatures are generally lower than in the gyre, but where no big turtle was released), this part of the analysis was restricted to locations east of 180°E only. The group of largest turtles was experiencing a wider range of SST and tended to be in warmer surface waters (mean SST of 19.0°C) than the small individuals (mean SST of 17.6°C for the first group).

**Figure 4:**
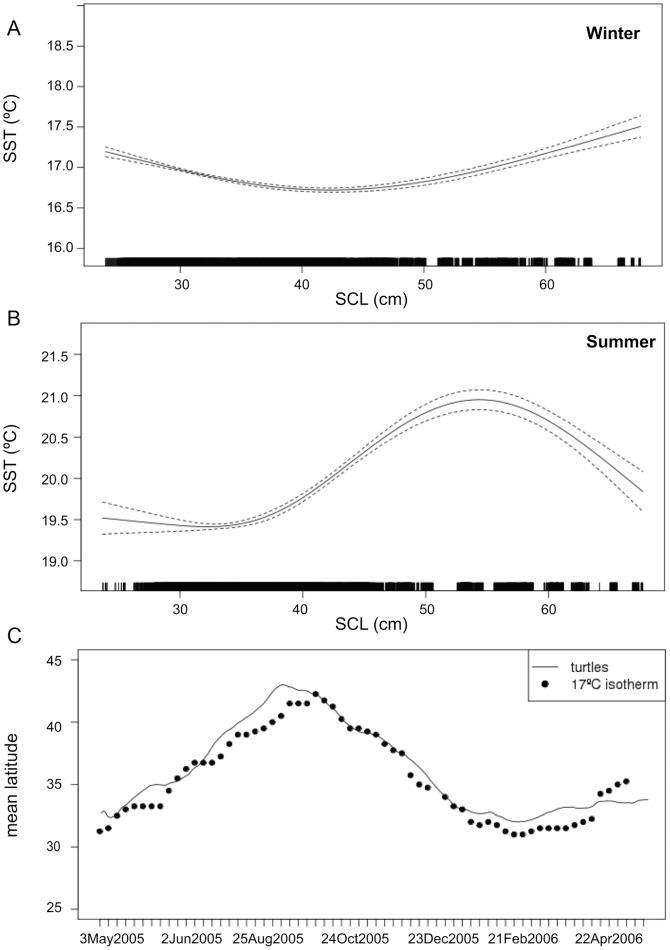
Relationship between SST and size of loggerhead turtles. **Smooth (solid line) and 95% confidence interval (dashed lines) of SST vs.** SCL estimated by the GAMs during A) the winter (Dec. to Mar.) and B) the summer (Jun. to Sept.). Ticks on the bottom axis represent values of SCL for which there is data; C) Mean latitude of observed turtles (29–38 cm, solid line) and of the 17°C SST isotherm at 190E longitude (black dots).

The potential impact of seasonal variability then was explored with two different GAMs, considering winter (December-March, when the surface layer is not stratified) and summer (June-September, when stratification is strong) separately and including SCL, the inter-annual (year) variation, as well as latitude and longitude, to account for other sources of variability in SST. An interesting difference appears in the relationship between SST and loggerhead size (Fig. 4). While there is not much difference in the SST encountered by big or small turtles in the winter (SST range: 16.7–17.5°C), the contrast is stronger in the summer (SST range: 19.5–21°C). To test whether this difference might be based on less active swimming capability by smaller turtles to keep up with oceanic changes to maintain themselves at their preferred temperature, the observed mean latitude of the turtles released in May 2005 (mean size: 35 cm) was compared with the mean latitude of the 17°C SST isotherm at 190°E longitude (Fig. 4). The close match of data suggests that juvenile turtles very likely have the capability to stay within a desirable range of temperature.

### Maximum Sustainable Speed (MSS)

Once the ocean currents speed was removed from the animals’ velocity, we were able to study the animals’ swimming speed (V = sqrt(V_x_?2+V_y_?2), with V_x_ and V_y_ the meridional and zonal components of the velocity vectors, respectively). However, as relative errors on the estimation of the current speeds are about constant [48], the non-random distribution of the turtles-with the smaller ones released in the highly dynamic Kuroshio current region and the bigger ones in the central gyre-could be problematic. To remain conservative, only locations east of the dateline, where currents and their estimation errors are relatively weak, were considered (62% of the data).

**Figure 5:**
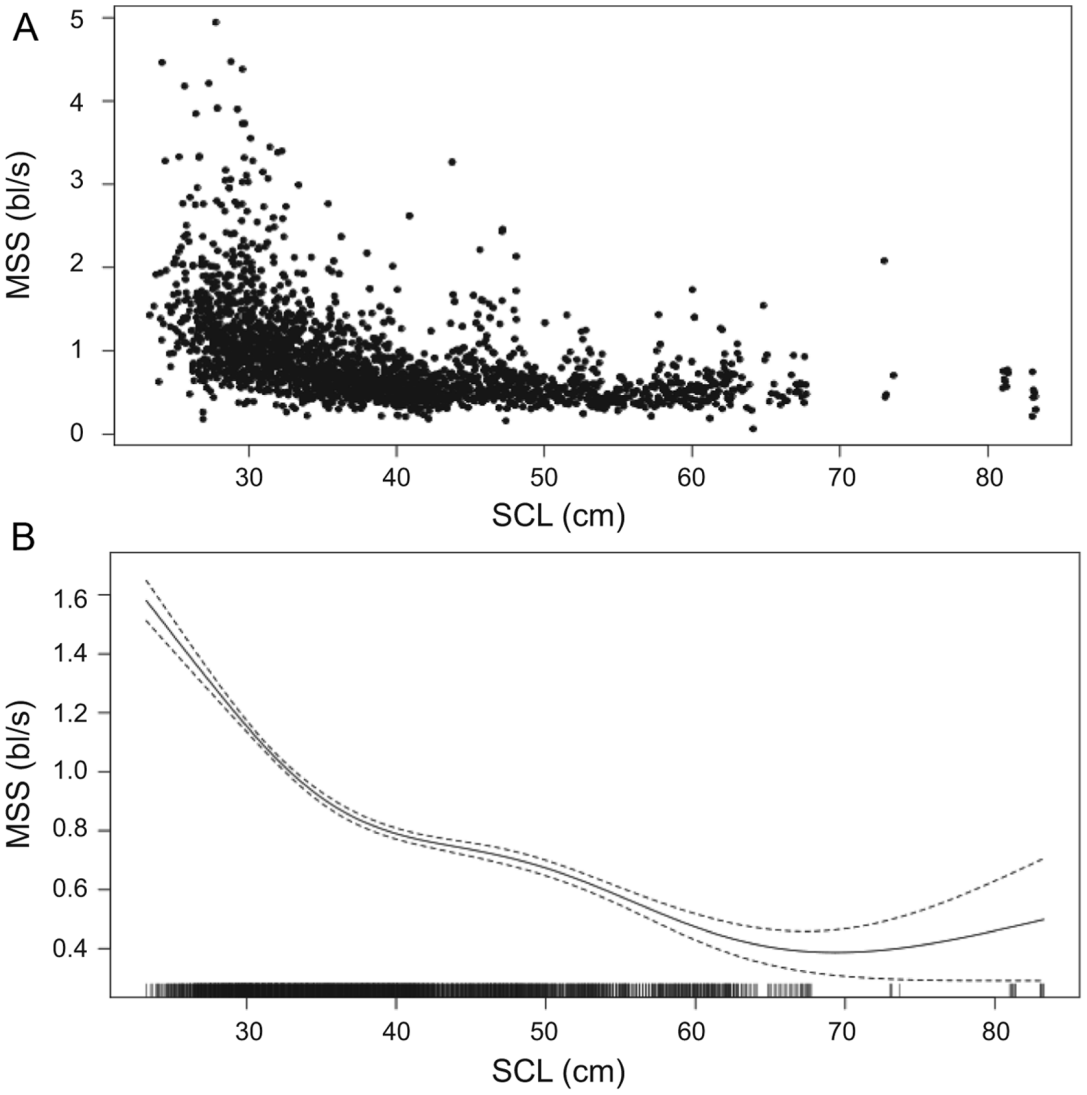
Relationship between speed and size of loggerhead turtles. A: Observed maximum sustainable speed (MSS, bl/s) against SCL (cm). B: smooth (solid line) and 95% confidence interval (dashed lines) of MSS vs. SCL estimated by the GAM. Ticks on the bottom axis represent values of SCL for which there is data.

Characteristic values of V for different size groups are presented in Table 3. It is interesting to note that even the smallest turtles exhibit non-zero speeds indicating that they do not always drift passively at those sizes. Mean swimming speeds for all size groups were under 1 km/h and increased with size, from 0.58 to 0.69 km/h, for turtles larger than 30 cm. Mean swimming speed for turtles smaller than 30 cm (0.61 km/h) was slightly higher than for medium-size turtles (Table 3). These values are of the same order of magnitude but generally lower than those estimated by [49] as critical swimming speeds for turtles ranging between 26 and 48 cm, which is to be expected for average travel swimming speeds. Despite a different definition used, our values of MSS (Fig. 5) are within the same range as the values of experimental critical swimming speed measured by [49].

**Table 3:** Values of swimming speeds (V, km/h) for different size groups.

size	Range of V	Mean V	Median V
<30 cm	0.02–7.54	0.61	0.56
30–40 cm	0.00–5.04	0.58	0.54
40–60 cm	0.01–8.56	0.59	0.53
> = 60cm	0.01–5.47	0.69	0.64
AOML drifters	0.00–8.36	0.69	0.58

MSS expressed in bl s^−1^ exhibited a decreasing relationship with SCL (Fig. 5A). Using the same approach as above, we ran a GAM to quantify the variation of MSS with SCL and other factors (longitude, latitude, month and year): 35% of the variability in MSS could be explained by SCL, and the smooth from the full model (Fig. 5B) allowed us to identify the value of MSS at a given size. The relationship suggests clearly that MSS rapidly decreases from about 1.6 to 0.5 bl s^−1^ for individuals under 67 cm SCL and then stabilizes around 0.5 bl s^−1^ for larger individuals with larger confidence intervals due to the sparsity of data for larger sizes. We also looked at the relationship between MSS and SST within a GAM, but SST only explained 0.5% of the variability.

### Feeding Habitat H_a_

**Figure 6:**
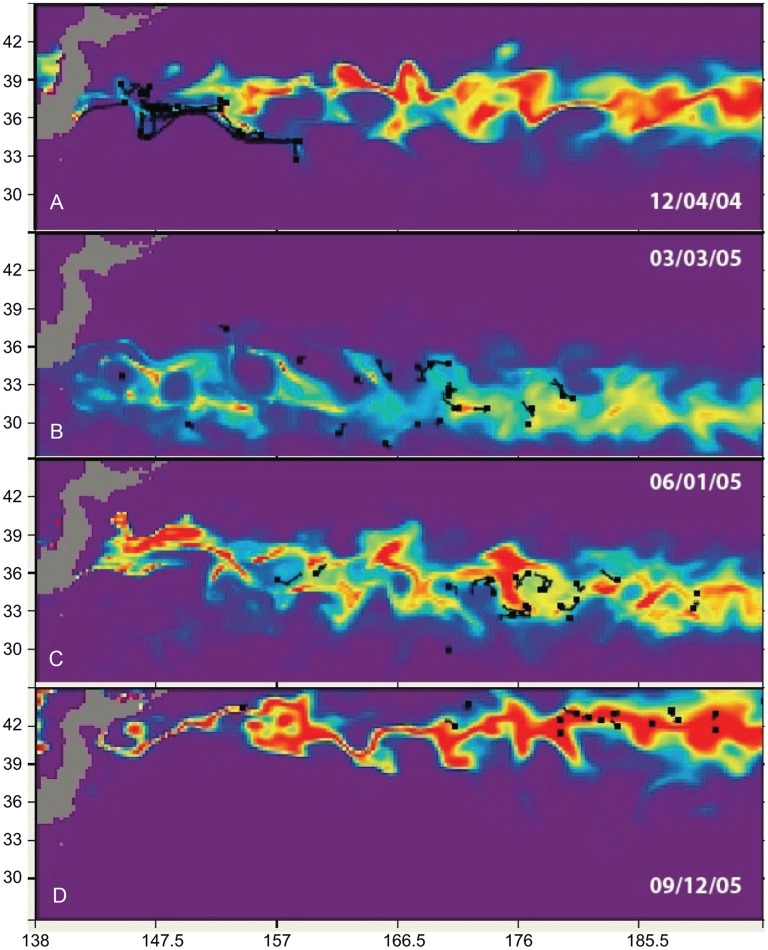
Modeled habitat index for November 2004 release. The habitat index (color scale, between 0 and 1) is overlaid with portions of tracks (black segments). From A to D: Dec. 4, 2004; Mar. 3, 2005; Jun. 1, 2005; Sep. 12, 2005.

**Figure 7:**
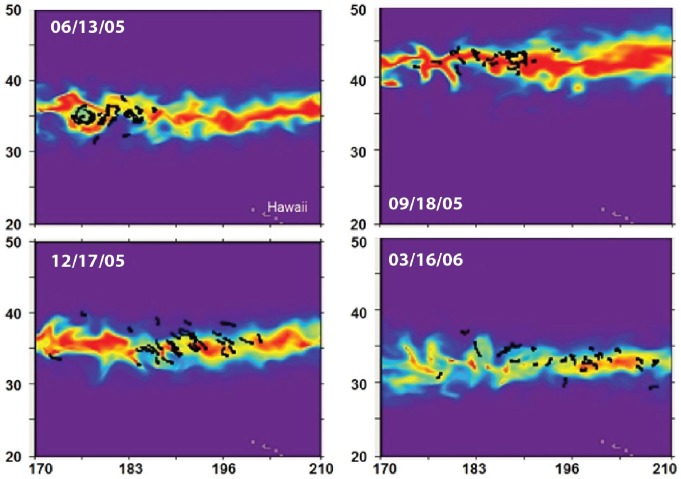
Modeled habitat index for May 2005 release. The habitat index (color scale, between 0 and 1) is overlaid with portions of tracks (black segments). From top to bottom: Jun. 13, 2005; Sep. 18, 2005; Dec. 17, 2005; Mar. 16, 2006.

Based on the previous analysis of temperature preference for a given size, we simulated the predicted habitat index for the November 2004 and the May 2005 releases (Table 2) in MOVEMOD, using the average of the turtle sizes for each batch. We used a mean temperature of 17°C for both releases, estimated from Fig. 4A. We postulated that in the absence of stratification, the animals would stay within a temperature range close to their optimal preference, which would then be around 17°C for all sizes. The standard deviations of SST were estimated from the tracks (Table 4). Figures 6 & 7 show snapshots, at quarterly intervals, of the predicted habitat index (*H_a_*) overlaid with the portions of tracks corresponding to the same 6-day (time step of the physical forcing) period. *Ha* is indicated by a color scale between 0 and 1, with 1 being optimal habitat (optimal temperature and abundance of prey), and 0 being least favorable. The areas with higher values of *H_a_* thus define areas of higher probability of presence. Figures 6 & 7 indicate that the simulated *H_a_* tracks the real turtles trajectories and their seasonal changes closely. In some of the snapshots, *H_a_* also presents eddy-like features that match the tracks, which provides confidence in the forcing used for this study.

**Table 4:** Parameters used for the simulations.

release	Average size	MSS^1^	Mean SST^2^	Std SST
November 2004	30.0	1.1	17.0	1.5
May 2005	34.7	0.9	17.0	1.5

1 Maximum Sustainable Speed.

2 Sea Surface Temperature.

**Figure 8:**
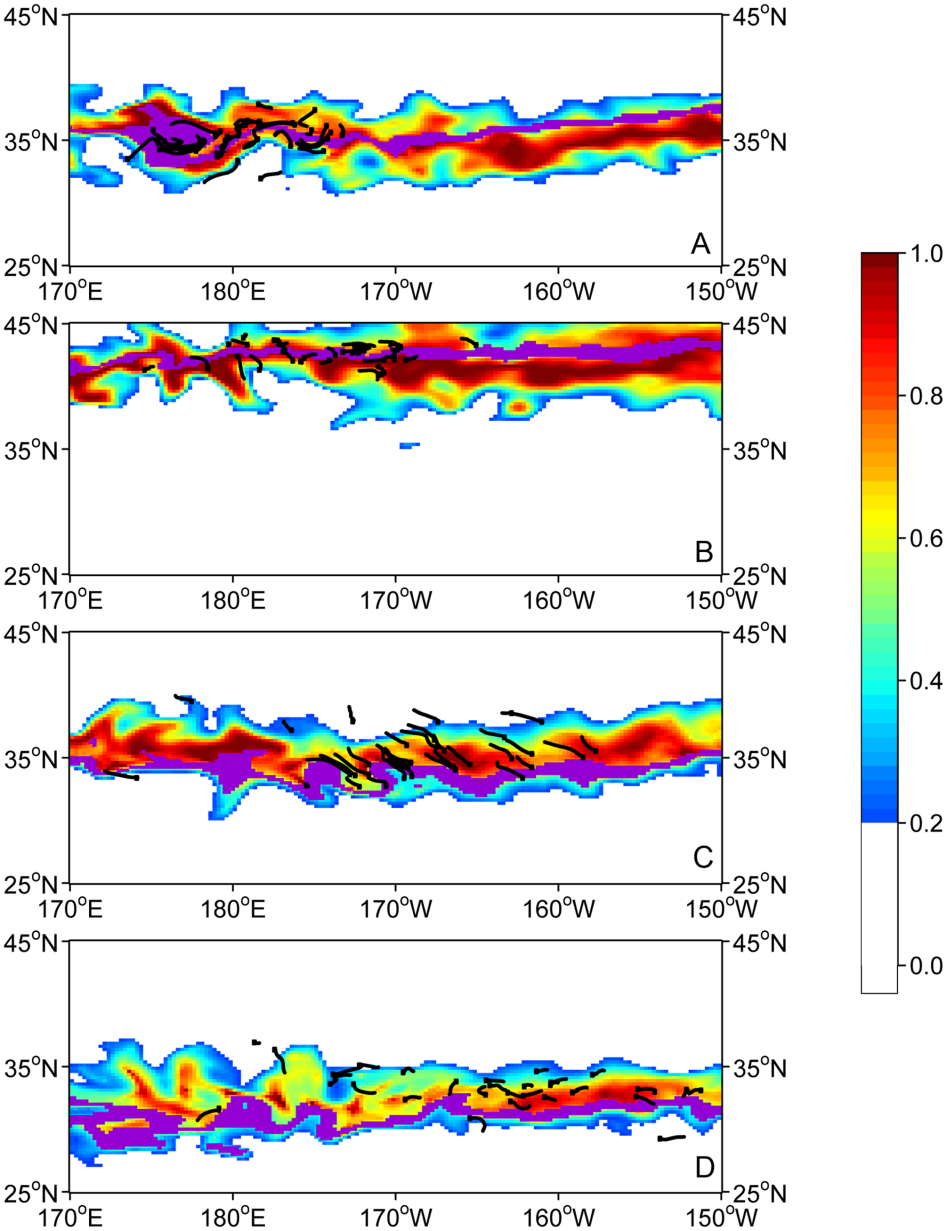
Comparison between the modeled habitat index and TurtleWatch. The habitat index (color scale, between 0 and 1) is overlaid with the TurtleWatch region (purple area) for the May 2005 release. From top to bottom: Jun. 13, 2005; Sep. 18, 2005; Dec. 17, 2005; Mar. 16, 2006

**Figure 9:**
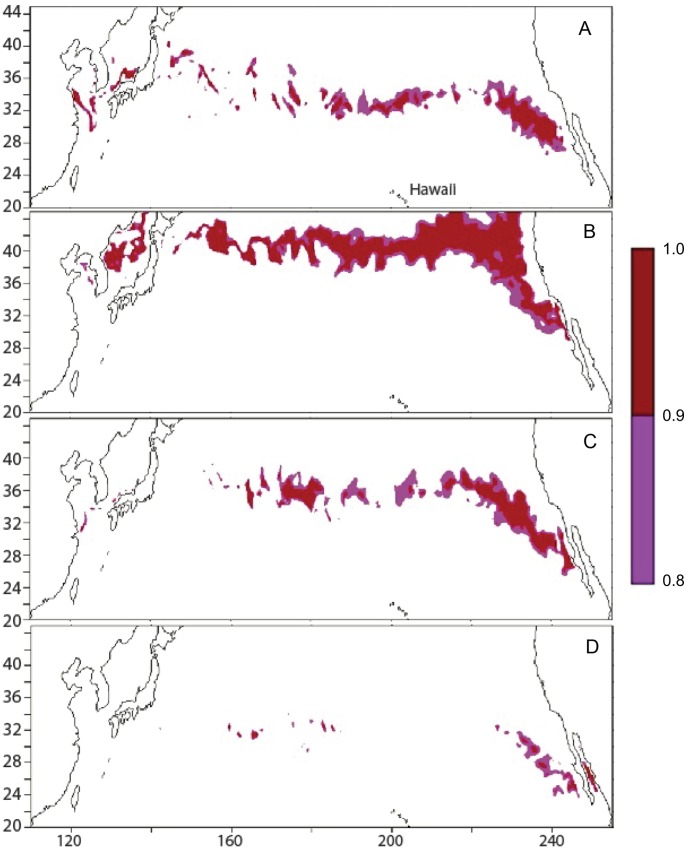
Predicted hot spots of habitat. Hot spots are defined as *H_a_* >0.8, for the May 2005 release. From A to D: Jun. 1, 2005; Sep. 12, 2005; Dec. 5, 2005; Mar. 4, 2006.

Although based on a very different approach, it is interesting to compare our habitat index with the NOAA TurtleWatch product [23]. Figure 8 illustrates how both indices compare for the May 2005 release. Both indices define roughly the same general area with a narrower latitudinal extension for the TurtleWatch index, usually centered on the maximum values of the habitat index *H_a_.* By setting a threshold to increasing values of *H_a_*, the habitat index can be restricted to increasingly favorable habitat, to identify hot spots of highest probability of turtle presence (Fig. 9), and corresponds to a closer match with TurtleWatch, but not always. In September, a maximum discrepancy between the two indices occurs, especially in the central Pacific, with *H_a_* being more to the south of TurtleWatch, while still in good agreement with the observed tracks (Figs. 6 & 7).

The maximum extension of most favorable habitat for 35cm-long juvenile loggerheads in the North Pacific (Fig. 9) is predicted to occur around September, with a broad area of high *H_a_* across the full basin length, and especially in the Eastern Pacific. Then, the overall extension of predicted hot spot areas is contracting during winter, reaching a minimum in March, with only a few remaining favorable hot spots off the coast of Baja California. During spring and summer, the extension of favorable areas expands again, reaching its maximum in September. Another important hot spot can be identified in the summer in the East China Sea.

### Movements

**Figure 10:**
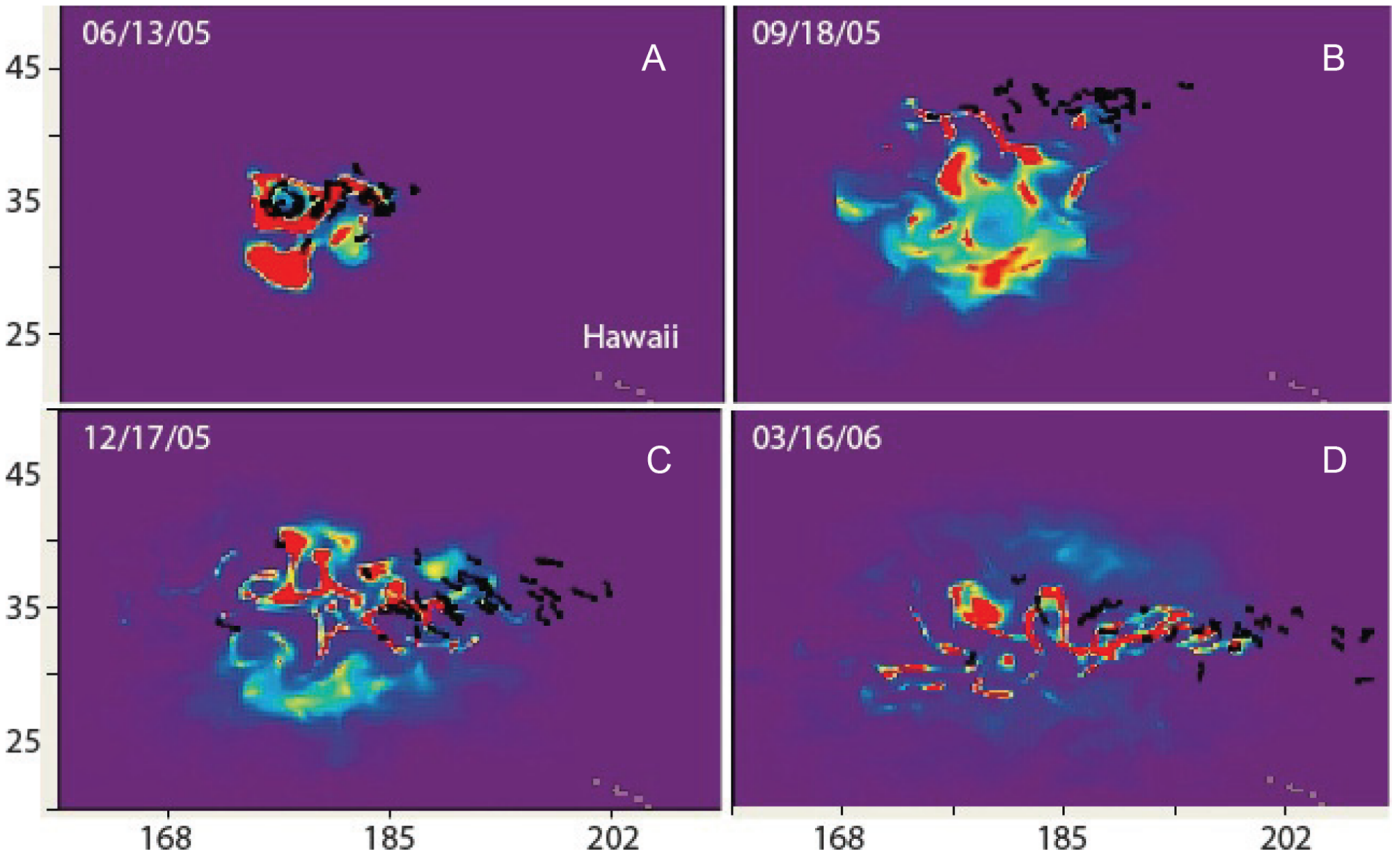
Modeled movements for May 2005 release. The density of turtles (# ind./cell, color scale) simulated with the habitat and movement model is overlaid with portions of tracks (black segments). From A to D: Jun. 13, 2005; Sep. 18, 2005; Dec. 17, 2005; Mar. 16, 2006.

**Figure 11:**
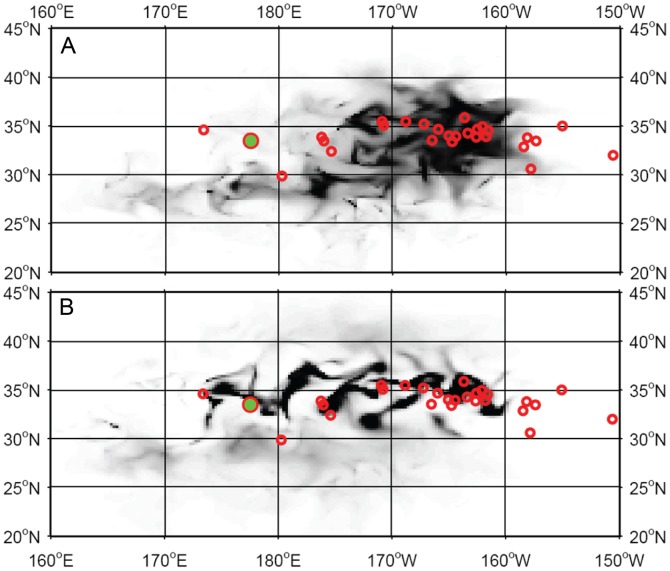
Comparison between active swimming and passive drift. Final predicted density distributions (gray scale) and observed individual locations (red circles) are compared between simulations using passive drift in ocean currents only (A) or combining directed movement and drift in ocean currents (B) in the movement equations. The green dot represents the release location of both real and simulated turtles.

Once the habitat was parameterized (Table 4), turtle movements were simulated with MOVEMOD using MSS values at size from previous analyses. Simulated turtles were released at the same location and date as the observed ones and predicted turtle densities were compared with observed tracks and final locations at quarterly intervals during 1-year of displacements. Following the movement mechanisms defined in the model [31], simulated turtles are pushed to move along gradients of the habitat index to leave poor habitat areas and move towards high habitat values. However, these rules of movement are also affected by ocean currents. Fig. 10 shows four similar snapshots of the simulated turtle density overlaid with the observed tracks for the May 2005 release, and Fig. 11 shows the simulated density at the end of one year, when the full movement equation is used, and when only passive drifting is considered, overlaid with the final locations of the released turtles. Density is expressed in number of individuals per 0.25°×0.25° cell but actual values are relative to the initial number released, which was arbitrarily fixed at 2000. There is generally a good match between the simulated density and the tracks, except in September when the observed turtles went farther north, even though *H_a_* in September was in good agreement with the tracks (Fig. 7). Some turtles also went farther east than the simulated ones.

As suggested by Fig. 5, even the smallest turtles in our dataset exhibited some active movement. Fig. 11 illustrates to what extent this affects the animals’ movements for the May 2005 release. When combining advection by ocean currents with the directed movements of the animals (Fig. 11B), the predicted turtle density after a year matched the observed turtle locations significantly better than when pure drift only is considered (Fig. 11A) and the concentrations of turtles were generally better represented.

## Discussion

Knowledge about the biology and ecology of juvenile loggerhead turtles is extremely limited based on the challenge of sampling such populations in the open ocean. In this context, the various deployments and tracking of such a significant number of juveniles provided the most comprehensive dataset in the Pacific Ocean, allowing us to investigate this poorly described life stage. However, some caution has to be taken since the turtles from the Japanese releases were reared in captivity. We believe they constitute an acceptable proxy to infer wild behaviors, assuming that reared turtles possess some innate sense from birth. No study to date has observed obvious oddities in migration or swimming behaviors of captive-reared turtles [10], [21], [25], [29]–[32], [50]. Nevertheless, further simulations comparing the habitat and behavior of wild and reared turtles with the same physical forcing would be valuable to confirm the results described in the present study.

The exceptional duration of the tracks in this study, with 59 individual tracks longer than one year and 14 longer than 2 years, raised the concern of accounting for growth during the time at liberty. Since there is no information on the growth of Pacific loggerhead turtles of medium to large sizes (>42 cm SCL, [51]), the growth rate used in this study was taken from recent North Atlantic estimates (Scott et al. 2011). However it is similar to growth rates in the North Pacific for small (<42 cm SCL) turtles [14], [51]. Questions remain as growth rates could vary between both areas, but given the scarcity of available information and the long durations of our tracks, using the North Atlantic relationship seemed more appropriate than using only the size at release for our analyses.

### Temperature

In ectothermic species, colder optimal temperatures and wider temperature ranges should be expected for larger individuals, since their larger size should increase their internal body temperature by reducing heat diffusivity [52].

The range of surface temperatures experienced by the bigger turtles in our dataset (SCL >50 cm) was larger than that experienced by smaller turtles (SCL<35 cm, Fig. 3), as would be expected, but the smaller turtles tended to be in colder surface waters than the bigger ones (Fig. 3), which is surprising. However, when considering winter and summer separately, coinciding respectively with weak (mixed-layer deeper than 40m) and strong (mixed layer between 10 and 40 m deep) stratification of the water column, small and large turtles remained in the same narrow range of temperature (16.7–17.5°C) in the winter, but were in two slightly different ranges in the summer (roughly 19–20 and 20–21°C, respectively, Fig. 4). We could assume that, when stratification is strong, turtles can quickly reach cold subsurface waters below the mixed-layer and thus could benefit from higher surface temperatures for rewarming. The observed higher SST values associated with larger individuals also concord with the idea that bigger turtles likely dive deeper than small ones and thus may target warmer surface waters. In winter however, when the mixed layer is too deep, all turtles would stay in a temperature range close to their optimal preference, which would be consequently around 17°C.

Unfortunately, the relationship between diving depth and size has not yet been investigated in details from electronic tracking data. The study by [32], based on a subset (*n* = 17) of the animals used in this present study that were equipped with depth sensors, did not allow such an analysis since all those individuals were in a relatively narrow range of sizes (43.5–66.5 cm). No significant relationship between the size of the turtles and the number or duration of the dives was found (E. Howell, pers. comm.). That group of juvenile loggerheads spent more than 80% of their time at depths above 5 m and dove at depths between 30 and 70 m, but remained in water temperatures warmer than 15°C. Another study [53] showed that in the Mediterranean Sea loggerhead dives over a large temperature range were sometimes linked to extended surface time, suggesting a rewarming function. Thus, the apparent preference of bigger turtles for warmer SSTs might be explained by differences in the diving depths with size, but further studies need to confirm this idea.

### Feeding Habitat

**Figure 12:**
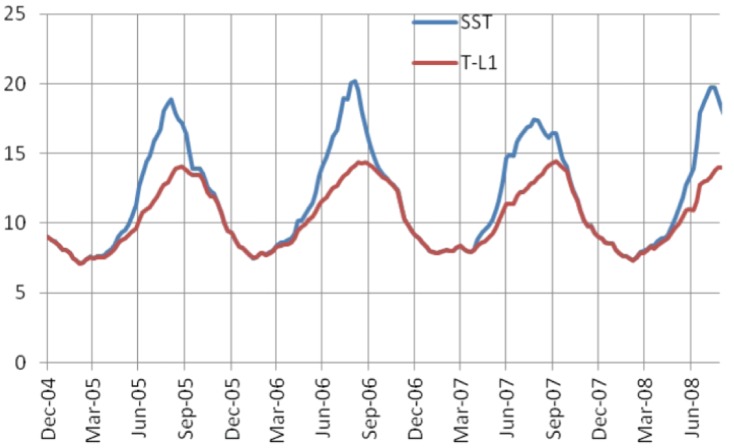
Comparison between the temperature at the surface and in the euphotic layer. SST (in °C, blue line) and average temperature of the euphotic layer (noted as T-L1, in °C, red line) from Dec. 2004 to Sept. 2008.

Loggerhead turtles at the oceanic stage in the central North Pacific feed mostly on neustonic (associated to the surface) prey items, such as *Carinaria cithara, Janthina spp., Lepas spp.,* and *Velella velella* [54], but only forage comprised in the whole epipelagic layer is available to date in SEAPODYM, which we used as a first approximation to describe loggerhead turtles’ foraging preference. However, it will be interesting for further analyses to develop a new functional group in SEAPODYM representing neustonic organisms. Based on the mechanisms of the model [47], we can expect a higher amplitude in the temperature cycle of the neustonic layer (i.e., the first 5 m) as well as stronger average currents with slightly different directions, thus leading to differences both in the time of development of organisms and their distribution. Interestingly, the largest discrepancy between habitat distribution and observed tracks occurs from August to early September (Fig. 10) in the most northern range of the habitat (40°-45°N), which is the period during which the upper ocean is most stratified and hence the period during which the SST differs most from the average temperature of the euphotic layer, used for the simulation of the epipelagic component of the micronekton (Fig. 12). Despite this limitation, our approach resulted in the definition of a habitat index that matches the tracks very closely, suggesting that temperature is the predominant factor to define accessibility to forage species, the interaction of both parameters driving the large scale movements of the species.

Habitat hot spots resulting from these interactions emerged from the simulations (Fig. 9). One permanent hot spot is located off the coast of Baja California. This area has been identified for a long time as a major feeding ground of juvenile loggerhead turtles [7], [11], [32], [55]–[59]. A genetics study [7] showed that 95% of sampled turtles along the coast of Baja California originated from Japanese nesting areas. The modeled habitat (Figs. 6, 7, & 9) predicts a clear transpacific route of migration between these two regions that is consistent with these observations. In addition, the maximum extension and favorability of this migration corridor occurs in Sep-Oct (Fig. 9) which coincides with the period of the year when hatchlings would leave nesting beaches after 2–3 months of incubation following the peak of the nesting season between the end of June and the beginning of July, in Japan [60], [61].

[10] identified the Kuroshio Extension Bifurcation Region (KEBR), between 155 and 180°E (ie., between Japan nesting grounds and Baja California foraging grounds), as a forage hot spot for loggerhead turtles because of the high primary production (PP) in that highly dynamic region during the fall, winter and spring, which also makes it a hot spot for various species [62]–[64]. That region was predicted to be a semi-permanent hot spot by our model but was not predicted to be an area as highly favorable as the Baja California hot spot. It is probably, however, a key area to support high survival rates of juveniles. This hot spot might also be underestimated by the model because of the definition of the epipelagic forage component in SEAPODYM, as discussed above. Although that area exhibits high PP, the development time defined for the epipelagic micronekton organisms considered as prey in this version of the model might be too long and cause the biomass of forage to be advected eastward too rapidly by the strong Kuroshio current. For the same reason probably, the hot spot in the East China Sea identified by [50] was identified as a hot spot by our model, but only during the summer (Fig. 9).

The feeding habitat index proposed here does not take into account any information from the longline fishery as was done to define the TurtleWatch index, but is purely based on temperature preferences and subsequent forage accessibility. The loggerhead turtles from the tracking data in [23] occurred most frequently in temperatures of 15.6 to 17.1°C, which is cooler than where the fisheries interactions mainly occurred (between 17.5 and 18.5°C). Given this discrepancy between the turtle distribution and that of the fisheries interactions, it is not surprising that the high *H_a_* values do not exactly match the TurtleWatch region. However, although they are not directly comparable, both indices predict the same general areas of highest probability of encountering loggerhead turtles and thus, areas with high risk of bycatch, in the North Pacific. An exception occurs around September, when the feeding habitat seems to peak at its maximum extension all over the North Pacific. At that time the TurtleWatch index seems to characterize the most northern limit of the habitat and could miss the main areas of concentration south of this limit. However, since the Hawaiian longline fishery mainly operates during the first and second quarters of the year, the TurtleWatch index does not really apply to this period of the year.

The feeding habitat index allows us to characterize the whole areas of highest probability of presence of loggerhead turtles year-round, and is independent of fisheries data. An analysis combining this feeding habitat and Pacific swordfish longline catches will provide a first evaluation of the idea of using this new index to constrain fishing so as to reduce bycatch of loggerhead turtles not only in Hawaiian waters but in the entire Pacific.

### Movements

A more quantitative way to measure the accuracy of the match between observed and predicted movements needs to be developed. For example, a tool calculating the values of predicted habitat index or density at each location along the observed tracks would help evaluate the performance of each simulation. Nonetheless, the present study suggests that even the smallest turtles in our data set (25 cm SCL) exhibit some level of active swimming, with mean swimming speeds around 0.6 km/h. We also used the AOML drifter database (http://www.aoml.noaa.gov/phod/dac/dacdata.php) to select drifter tracks for comparison with the turtles. The same analysis was performed on the drifter tracks to remove currents speed and a Kolmogorov-Smirnov test was performed to compare the turtles and drifters’ “swimming” speeds. The test indicated significant differences between turtle and drifter behaviors which supports our interpretation that turtles are not passively drifting but their movements likely incorporate behavior. This is valuable information as young turtles are rarely observed in the wild, and very little is known about their behavior and abilities. The oceanic stage between the departure from hatching to the return to neritic areas (around 70 cm in size) many years later has been called “the lost years” [65] in reference to this lack of observability. Most tracking studies on wild turtles have been conducted on larger animals, either around nesting areas or caught in the various fisheries [10], [66]–[69].

[70] performed simulations of loggerhead hatchling dispersal from Japanese nesting beaches using only passive drifting in the movement and studied the extent of their eastward drift. No particle reached Baja California within 5 years in their simulations. Sixty-five of the turtles in our dataset were released off Japan, only 16 of which had track durations of at least 1 year (with SCL at the time of release between 24 and 65 cm), and one of which had a track duration of more than 2 years. At one year after release, 62.5% of those 16 turtles were between 180 and 200°E vs. only 46.6% of the simulated hatchlings in [70] and 6.3% were between 200 and 220°E vs. 0.2%. Only one turtle (SCL = 36.5 cm) in our dataset, which was released in the Central Pacific at 176.6°E longitude, reached the coast of Baja California after 4.9 years (Fig. 1B).

We chose the November 2004 and the May 2005 releases to illustrate our modeling approach because their ranges of sizes were small enough to reasonably treat all the turtles released on each day as one single age cohort. The May 2005 release is also of particular interest because it was conducted in the area of operation of the Hawaii-based shallow-set longline fishery.

The observed tracks seem to be more concentrated in areas of high predicted habitat index (*H_a_*) when *H_a_* reaches its peak in September. Conversely, when *H_a_* reaches minimum values basin-wide in March, tracks appear much more dispersed (Fig. 6B). This pattern would tend to validate the approach used to describe the movement mechanisms proposed in the Eulerian framework of SEAPODYM, with increasing diffusion when habitat index values decrease and stronger advection (i.e., directed movements) linked to increasing gradients of habitat, both being proportional to the size of the animals [45]. Additionally, observed and predicted turtle locations after 1 year at liberty are clearly in better agreement when using these rules of movement (including also the impact of currents) than when considering pure drifting only, providing confidence in the modeling of both the habitat and the movements.

Nevertheless, the model still needs to be tested for other release experiments, especially with larger individuals. In that case, one issue will be to differentiate between feeding behavior and spawning migrations. A version of SEAPODYM that allows tracking data assimilation is being developed, which could help estimate more precisely the feeding habitat parameters. For reproductive migrations however, temperature and availability of prey would not be the only factors affecting the large-scale movements of loggerhead turtles. This modeling framework could be easily used to introduce and test various migration hypotheses, e.g. based on magnetic fields [71], [72].

Twenty-six turtles of our dataset, with SCL ranging between 35 and 83 cm, exhibited a net displacement of at least 1 degree westward between the first and last locations of their tracks while all others showed a net eastward displacement. However, our model does not reproduce those westward movements. This suggests that temperature and availability of prey are not the only factors affecting the large-scale movements of loggerhead turtles. Juvenile and sub-adult turtles spend decades in the open ocean undertaking foraging migrations. Those observed westward movements might be used to offset eastward transport and constitute retention behavior.

The oceanic stage of loggerhead turtles has been estimated to last several decades, while our longest track lasted about 3.5 years. Until tracking capabilities allow for datasets encompassing the entire oceanic stage, understanding the timing of the east-west dynamics of juvenile loggerheads will remain challenging. Nevertheless, confronting these individual tracks to dynamic habitat and movement models already reveals a promising development with direct potential applications that could be rapidly used for assisting in the conservation of these endangered species.

## References

[1] Crowder LB, Murawski SA (1998) Fisheries Bycatch: Implications for Management. Fisheries 23: 8–17 doi:;10.1577/1548-8446(1998)023<0008:FBIFM>2.0.CO;2

[2] Zydelis R, Lewison RL, Shaffer SA, Moore JE, Boustany AM, et al (2011) Dynamic habitat models: using telemetry data to project fisheries bycatch. Proc R Soc B Biol Sci 278: 3191–3200 doi:10.1098/rspb.2011.0330 10.1098/rspb.2011.0330 PMC3169031 21429921

[3] Hobday AJ, Hartog JR, Timmiss T, Fielding J (2010) Dynamic spatial zoning to manage southern bluefin tuna (Thunnus maccoyii) capture in a multi-species longline fishery. Fish Ocean 19: 243–253 doi:10.1111/j.1365-2419.2010.00540.x

[4] Block BA, Jonsen ID, Jorgensen SJ, Winship AJ, Shaffer SA, et al (2011) Tracking apex marine predator movements in a dynamic ocean. Nature 475: 86–90 doi:10.1038/nature10082 21697831 10.1038/nature10082

[5] Kamezaki N, Matsuzawa Y, Abe O, Asakawa H, Fuji T, et al.. (2003) Loggerhead turtle nesting in Japan. Loggerhead Sea Turtles. Smithsonian Books, Washington. 210–217.

[6] Limpus CJ, Limpus DJ (2003) Loggerhead turtles in the Equatorial and Southern Pacific Ocean: A species in decline. Loggerhead Sea Turtles. Smithsonian Books, Washington. 199–209.

[7] Bowen BW, Abreu-Grobois FA, Balazs GH, Kamezaki N, Limpus CJ, et al (1995) Trans-Pacific migrations of the loggerhead turtle (Caretta caretta) demonstrated with mitochondrial DNA markers. Proc Natl Acad Sci 92: 3731–3734. 7731974 10.1073/pnas.92.9.3731 PMC42035

[8] Bowen BW, Karl SA (2007) Population genetics and phylogeography of sea turtles. Mol Ecol 16: 4886–4907 doi:10.1111/j.1365-294X.2007.03542.x 17944856 10.1111/j.1365-294X.2007.03542.x

[9] Hatase H, Kinoshita M, Bando T, Kamezaki N, Sato K, et al (2002) Population structure of loggerhead turtles, Caretta caretta, nesting in Japan: bottlenecks on the Pacific population. Mar Biol 141: 299–305 doi:10.1007/s00227-002-0819-4

[10] Polovina J, Uchida I, Balazs G, Howell EA, Parker D, et al (2006) The Kuroshio Extension Bifurcation Region: A pelagic hotspot for juvenile loggerhead sea turtles. Deep Sea Res Part Ii Top Stud Ocean 53: 326–339 doi:10.1016/j.dsr2.2006.01.006

[11] Peckham S, Maldonado Diaz D, Tremblay Y, Ochoa R, Polovina J, et al (2011) Demographic implications of alternative foraging strategies in juvenile loggerhead turtles Caretta caretta of the North Pacific Ocean. Mar Ecol Prog Ser 425: 269–280 doi:10.3354/meps08995

[12] Baldwin R, Hughes G, Prince R, I T. (2003) Loggerhead Turtles in the Indian Ocean. Logg. Smithsonian Books, Washington. 218–232.

[13] Hughes G (2010) Loggerheads and leatherbacks in the Western Indian Ocean. Indian Ocean Turtle Newsletter. Vol. 11: 24–31.

[14] Scott R, Marsh R, Hays GC (2012) Life in the really slow lane: loggerhead sea turtles mature late relative to other reptiles. Funct Ecol 26: 227–235 doi:10.1111/j.1365-2435.2011.01915.x

[15] Witherington BE (2003) Biological Conservation of Loggerheads: Challenges and opportunities. Loggerhead Sea Turtles. Smithsonian Books, Washington. 295–311.

[16] Lewison RL, Crowder LB (2007) Putting Longline Bycatch of Sea Turtles into Perspective. Conserv Biol 21: 79–86 doi:10.1111/j.1523-1739.2006.00592.x 17298513 10.1111/j.1523-1739.2006.00592.x

[17] Ishihara T (2007) Japan coastal bycatch investigations. North Pacific Loggerhead Sea Turtle Expert Workshop December 19–20, 2007. Western Pacific Regional Fish- ery Management Council and U.S. National Marine Fisheries Service. 21–22.

[18] Ishihara T, Kamezaki N, Matsuzawa Y, Iwamoto F, Oshika T, et al (2011) Reentery of Juvenile and Subadult Loggerhead Turtles into Natal Waters of Japan. Curr Herpetol 30: 63–68.

[19] Gilman E, Brothers N, Kobayashi DR (2005) Principles and approaches to abate seabird by-catch in longline fisheries. Fish Fish 6: 35–49.

[20] Lewison RL, Freeman SA, Crowder LB (2004) Quantifying the effects of fisheries on threatened species: the impact of pelagic longlines on loggerhead and leatherback sea turtles. Ecol Lett 7: 221–231 doi:10.1111/j.1461-0248.2004.00573.x

[21] Swimmer Y, Brill R (2006) Sea Turtle and Pelagic Fish Sensory Biology: Developing Techniques to Reduce Sea Turtle Bycatch in Longline Fisheries.

[22] Gilman E, Kobayashi D, Swenarton T, Brothers N, Dalzell P, et al (2007) Reducing sea turtle interactions in the Hawaii-based longline swordfish fishery. Biol Conserv 139: 19–28 doi:10.1016/j.biocon.2007.06.002

[23] Howell E, Kobayashi D, Parker D, Balazs G, Polovina aJJ (2008) TurtleWatch: a tool to aid in the bycatch reduction of loggerhead turtles Caretta caretta in the Hawaii-based pelagic longline fishery. Endanger Species Res 5: 267–278 doi:10.3354/esr00096

[24] Benson S, Dewar H, et al. (2009) Swordfish and Leatherback use of temperate habitat (SLUTH). Available: http://www.pcouncil.org/bb/2009/0409/D1b_ATT1_0409.pdf. Accessed 10 April 2013.

[25] Polovina JJ, Howell E, Parker DM, Balazs GH (2003) Dive-depth distribution of loggerhead(Carretta carretta) and olive ridley(Lepidochelys olivacea) sea turtles in the Central North Pacific: Might deep longline sets catch fewer turtles? Fish Bull 101: 189–193.

[26] Beverly S, Robinson E, Itano D (2004) Trial setting of deep longline techniques to reduce bycatch and increase targeting of deep-swimming tunas. 17th Meeting of the Standing Committee on Tuna and Billfish, SCTB17, Majuro, Marshall Islands. 9–18. Available: http://www.spc.int/DigitalLibrary/Doc/FAME/Meetings/SCTB/17/FTWG_7a.pdf. Accessed 11 April 2013.

[27] Beverly S, Curran D, Musyl M, Molony B (2009) Effects of eliminating shallow hooks from tuna longline sets on target and non-target species in the Hawaii-based pelagic tuna fishery. Fish Res 96: 281–288.

[28] Abecassis M, Dewar H, Hawn D, Polovina J (2012) Modeling swordfish daytime vertical habitat in the North Pacific Ocean from pop-up archival tags. Mar Ecol Prog Ser 452: 219–236 doi:10.3354/meps09583

[29] Kobayashi DR, Polovina JJ, Parker DM, Kamezaki N, Cheng I-J, et al (2008) Pelagic habitat characterization of loggerhead sea turtles, Caretta caretta, in the North Pacific Ocean (1997–2006): Insights from satellite tag tracking and remotely sensed data. J Exp Mar Biol Ecol 356: 96–114 doi:10.1016/j.jembe.2007.12.019

[30] Polovina JJ, Kobayashi DR, Parker DM, Seki MP, Balazs GH (2000) Turtles on the edge: movement of loggerhead turtles (Caretta caretta) along oceanic fronts, spanning longline fishing grounds in the central North Pacific, 1997–1998. Fish Ocean 9: 71–82.

[31] Polovina JJ, Balazs GH, Howell EA, Parker DM, Seki MP, et al (2004) Forage and migration habitat of loggerhead (Caretta caretta) and olive ridley (Lepidochelys olivacea) sea turtles in the central North Pacific Ocean. Fish Ocean 13: 36–51.

[32] Howell EA, Dutton PH, Polovina JJ, Bailey H, Parker DM, et al (2010) Oceanographic influences on the dive behavior of juvenile loggerhead turtles (Caretta caretta) in the North Pacific Ocean. Mar Biol 157: 1011–1026 doi:10.1007/s00227-009-1381-0

[33] Balazs GH, Miya RK, Beaver SC, et al.. (1996) Procedures to attach a satellite transmitter to the carapace of an adult green turtle, Chelonia mydas. Proceedings of the Fifteenth Annual Symposium on Sea Turtle Biology and Conservation. Hilton Head, South Carolina. 21–26.

[34] Gaspar P, Georges J-Y, Fossette S, Lenoble A, Ferraroli S, et al (2006) Marine animal behaviour: neglecting ocean currents can lead us up the wrong track. Proc R Soc B Biol Sci 273: 2697–2702 doi:10.1098/rspb.2006.3623 10.1098/rspb.2006.3623 PMC1635505 17015330

[35] Seifert B, Gasser T (1998) Local polynomial smoothing. Encyclopedia of Statistical Sciences, Update. Wiley, Vol. 2: 367–372.

[36] R Development Core Team (2008) R: A language and environment for statistical computing. Vienna, Australia. Available: http://www.R-project.org.

[37] Hastie T, Tibshirani R (1990) Generalized Additive Models. Chapman and Hall, London, U.K.

[38] Wood S (2006) Generalized Additive Models: An Introduction with R. Chapman and Hall/CRC.

[39] Bernard B, Madec G, Penduff T, Molines J-M, Treguier A-M, et al (2006) Impact of partial steps and momentum advection schemes in a global ocean circulation model at eddy-permitting resolution. Ocean Dyn 56: 543–567 doi:10.1007/s10236-006-0082-1

[40] Tuan Pham D, Verron J, Christine Roubaud M (1998) A singular evolutive extended Kalman filter for data assimilation in oceanography. J Mar Syst 16: 323–340.

[41] Tranchant B, Testut C, Renault L, Ferry N, Birol F, et al (2008) Expected impact of the future SMOS and Aquarius Ocean surface salinity missions in the Mercator Ocean operational systems: New perspectives to monitor ocean circulation. Remote Sens Environ 112: 1476–1487 doi:10.1016/j.rse.2007.06.023

[42] Behrenfeld MJ, Falkowski PG (1997) Photosynthetic rates derived from satellite-based chlorophyll concentration. Limnol Ocean 42: 1–20.

[43] Lehodey P (2001) The pelagic ecosystem of the tropical Pacific Ocean: dynamic spatial modelling and biological consequences of ENSO. Prog Ocean 49: 439–468.

[44] Lehodey P, Chai F, Hampton J (2003) Modelling climate-related variability of tuna populations from a coupled ocean–biogeochemical-populations dynamics model. Fish Ocean 12: 483–494.

[45] Lehodey P, Senina I, Murtugudde R (2008) A spatial ecosystem and populations dynamics model (SEAPODYM) – Modeling of tuna and tuna-like populations. Prog Ocean 78: 304–318 doi:10.1016/j.pocean.2008.06.004

[46] Senina I, Sibert J, Lehodey P (2008) Parameter estimation for basin-scale ecosystem-linked population models of large pelagic predators: Application to skipjack tuna. Prog Ocean 78: 319–335 doi:10.1016/j.pocean.2008.06.003

[47] Lehodey P, Murtugudde R, Senina I (2010) Bridging the gap from ocean models to population dynamics of large marine predators: A model of mid-trophic functional groups. Prog Ocean 84: 69–84 doi:10.1016/j.pocean.2009.09.008

[48] Rio MH, Guinehut S, Larnicol G (2011) New CNES-CLS09 global mean dynamic topography computed from the combination of GRACE data, altimetry, and in situ measurements. J Geophys Res 116. Available: http://doi.wiley.com/10.1029/2010JC006505. Accessed 12 April 2013.

[49] Revelles M, Carreras C, Cardona L, Marco A, Bentivegna F, et al (2007) Evidence for an asymmetrical size exchange of loggerhead sea turtles between the Mediterranean and the Atlantic through the Straits of Gibraltar. J Exp Mar Biol Ecol 349: 261–271 doi:10.1016/j.jembe.2007.05.018

[50] Kobayashi DR, Cheng I-J, Parker DM, Polovina JJ, Kamezaki N, et al (2011) Loggerhead turtle (Caretta caretta) movement off the coast of Taiwan: characterization of a hotspot in the East China Sea and investigation of mesoscale eddies. Ices J Mar Sci 68: 707–718 doi:10.1093/icesjms/fsq185

[51] Zug GR, Balazs GH, Wetherall JA (1995) Growth in juvenile loggerhead seaturtles (Caretta caretta) in the north Pacific pelagic habitat. Copeia 1995: 484–487.

[52] Stevenson R (1985) Body Size and Limits to the Daily Range of Body Temperature in Terrestrial Ectotherms. Am Nat 125: 102–117.

[53] Hochscheid S, Bentivegna F, Hamza A, Hays GC (2010) When surfacers do not dive: multiple significance of extended surface times in marine turtles. J Exp Biol 213: 1328–1337 doi:10.1242/jeb.037184 20348345 10.1242/jeb.037184

[54] Parker DM, Cooke WJ, Balazs GH (2005) Diet of oceanic loggerhead sea turtles (Caretta caretta) in the central North Pacific. Fish Bull 103: 142–152.

[55] Resendiz A, Resendiz B, Nichols WJ, Seminoff JA, Kamezaki N (1998) First confirmed eastwest transpacific movement of a loggerhead sea turtle, Caretta caretta, released in Baja California, Mexico. Pac Sci 52: 151–153.

[56] Nichols WJ, Resendiz A, Seminoff JA, Resendiz B (2000) Transpacific migration of a loggerhead turtle monitored by satellite telemetry. Bull Mar Sci 67: 937–947.

[57] Etnoyer P, Canny D, Mate BR, Morgan LE, Ortega-Ortiz JG, et al (2006) Sea-surface temperature gradients across blue whale and sea turtle foraging trajectories off the Baja California Peninsula, Mexico. Deep Sea Res Part Ii Top Stud Ocean 53: 340–358 doi:10.1016/j.dsr2.2006.01.010

[58] Peckham SH, Diaz DM, Walli A, Ruiz G, Crowder LB, et al (2007) Small-Scale Fisheries Bycatch Jeopardizes Endangered Pacific Loggerhead Turtles. Plos One 2: e1041 doi:10.1371/journal.pone.0001041 17940605 10.1371/journal.pone.0001041 PMC2002513

[59] Peckham S, Maldonado Diaz D, Koch V, Mancini A, Gaos A, et al (2008) High mortality of loggerhead turtles due to bycatch, human consumption and strandings at Baja California Sur, Mexico, 2003 to 2007. Endanger Species Res 5: 171–183 doi:10.3354/esr00123

[60] Sato K, Bando T, Matsuzawa Y, et al (1997) Decline of the loggerhead turtle, Caretta caretta, nesting on Senri Beach in Minabe, Wakayama, Japan. Chelonian Conserv Biol 2: 600–603.

[61] Matsuzawa Y, Sato K, Sakamoto W, Bjorndal KA (2002) Seasonal fluctuations in sand temperature: effects on the incubation period and mortality of loggerhead sea turtle (Caretta caretta ) pre-emergent hatchlings in Minabe, Japan. Mar Biol 140: 639–646 doi:10.1007/s00227-001-0724-2

[62] Zainuddin M, Kiyofuji H, Saitoh K, Saitoh S-I (2006) Using multi-sensor satellite remote sensing and catch data to detect ocean hot spots for albacore (Thunnus alalunga) in the northwestern North Pacific. Deep Sea Res Part Ii Top Stud Ocean 53: 419–431 doi:10.1016/j.dsr2.2006.01.007

[63] Suryan RM, Sato F, Balogh GR, David Hyrenbach K, Sievert PR, et al (2006) Foraging destinations and marine habitat use of short-tailed albatrosses: A multi-scale approach using first-passage time analysis. Deep Sea Res Part Ii Top Stud Ocean 53: 370–386 doi:10.1016/j.dsr2.2006.01.012

[64] Okazaki Y, Nakata H, Kimura S, Kasai A (2003) Offshore entrainment of anchovy larvae and its implication for their survival in a frontal region of the Kuroshio. Mar Ecol Prog Ser 248: 237–244.

[65] Bolten AB, Balazs GH (1995) Biology of the early pelagic stage–the “lost year.” Biology and conservation of sea turtles. Smithsonian Institution Press, Washington D.C., USA. 579–581.

[66] Avens L, Braun-McNeill J, Epperly S, Lohmann KJ (2003) Site fidelity and homing behavior in juvenile loggerhead sea turtles (Caretta caretta ). Mar Biol 143: 211–220 doi:10.1007/s00227-003-1085-9

[67] Eckert SA, Moore JE, Dunn DC, van Buiten RS, Eckert KL, et al (2008) Modeling loggerhead turtle movement in the Mediterranean: importance of body size and oceanography. Ecol Appl 18: 290–308. 18488597 10.1890/06-2107.1

[68] Revelles M, Cardona L, Aguilar A, San Félix M, Fernández G (2007) Habitat use by immature loggerhead sea turtles in the Algerian Basin (western Mediterranean): swimming behaviour, seasonality and dispersal pattern. Mar Biol 151: 1501–1515 doi:10.1007/s00227-006-0602-z

[69] Revelles M, Isern-Fontanet J, Cardona L, San Félix M, Carreras C, et al (2007) Mesoscale eddies, surface circulation and the scale of habitat selection by immature loggerhead sea turtles. J Exp Mar Biol Ecol 347: 41–57 doi:10.1016/j.jembe.2007.03.013

[70] Okuyama J, Kitagawa T, Zenimoto K, Kimura S, Arai N, et al (2011) Trans-Pacific dispersal of loggerhead turtle hatchlings inferred from numerical simulation modeling. Mar Biol 158: 2055–2063 doi:10.1007/s00227-011-1712-9

[71] Lohmann KJ, Lohmann CM, Ehrhart LM, Bagley DA, Swing T (2004) Animal behaviour: geomagnetic map used in sea-turtle navigation. Nature 428: 909–910. 15118716 10.1038/428909a

[72] Lohmann KJ, Luschi P, Hays GC (2008) Goal navigation and island-finding in sea turtles. J Exp Mar Biol Ecol 356: 83–95 doi:10.1016/j.jembe.2007.12.017

